# A Hybrid Bidirectional Deep Learning Model Using HRV for Prediction of ICU Mortality Risk in TBI Patients

**DOI:** 10.1007/s41666-025-00209-5

**Published:** 2025-07-30

**Authors:** Hasitha Kuruwita A., Shu Kay Ng, Alan Wee-Chung Liew, Kelvin Ross, Brent Richards, Kuldeep Kumar, Luke Haseler, Ping Zhang

**Affiliations:** 1 School of Medicine and Dentistry, Griffith University, Gold Coast, Australia; 2 School of Medicine and Dentistry, Griffith University, Nathan, Australia; 3 School of ICT, Griffith University, Gold Coast, Australia; 4 Datarwe, Gold Coast, Australia; 5 IntelliHQ, Gold Coast, Australia; 6 Bond University, Gold Coast, Australia; 7 Curtin School of Allied Health, Curtin University, Perth, Australia

**Keywords:** Traumatic brain injury, Heart rate variability, Mortality prediction, Machine learning, Clinical decision support

## Abstract

Accurately predicting early mortality risk for traumatic brain injury (TBI) patients admitted to the intensive care unit (ICU) is crucial for optimizing patient care, allocating resources effectively, and reducing mortality rates. This study introduces an approach to predict mortality risk for TBI patients by analysing heart rate variability from the first 24 h of electrocardiogram (ECG) signals. A deep learning hybrid model was developed by integrating a weight predictor with a bidirectional long short-term memory (BiLSTM) unit. This hybrid architecture enhances predictive performance by weighting features and capturing patterns in HRV data. This study utilised TBI patient data from the Gold Coast University Hospital and Cerebral Haemodynamic Autoregulatory Information System (CHARIS) for model training and testing. The experimental results demonstrated that the proposed hybrid model achieved cross-validation metrics, including an accuracy of 0.933 (95% CI: 0.844–1.000), an area under the curve of the receiver operating characteristics (AUROC) of 0.995 (0.978–1.000), and an area under the precision‒recall curve (AUPRC) of 0.998 (0.99–1.000). With the hold-out test dataset, the model obtained a prediction accuracy of 0.917 (0.75–1.000), an AUROC of 0.926 (0.766–1.000), and an AUPRC of 1.0. Comparative analysis with conventional machine learning models confirmed that the proposed model significantly outperformed existing approaches. The results highlight the potential of the proposed model in helping critical care strategies by providing more accurate early predictions of mortality risk through HRV analysis. Since the proposed model relies exclusively on ICU monitoring ECG data, it facilitates straightforward implementation in clinical settings.

**Supplementary Information:**

The online version contains supplementary material available at 10.1007/s41666-025-00209-5.

## Introduction

Traumatic brain injury (TBI) patients require specialised care in the intensive care unit (ICU) because of their high mortality risk and the complex challenges associated with the management of their condition compared with other hospitalised patients [1]. Managing TBI cases is complicated by the severity of the injury and related consequences, such as intracranial hemorrhage and secondary brain damage [2]. Globally, it is estimated that between 27 and 69 million people require intensive care treatment annually after brain injury [3], and approximately 30% of ICU-admitted brain injury patients do not survive their stay [4, 5]. Accordingly, accurate mortality risk prediction is essential to optimise patient care in a timely manner, allocate ICU medical resources effectively, and improve patients’ outcomes. Various models have been developed over the years using admission data. However, these models are not capable of capturing the dynamic, time-evolving nature of patients’ physiological responses to injury and treatment as they evolve over time. In addition, these models are limited by their reliance on electronic medical records (EMRs), which may be incomplete, unavailable in certain hospitals, and not immediately available or unreliable. Similarly, traditional scoring systems have drawbacks such as subjectivity, an inability to address the heterogeneity of injuries, and low predictive power [6–9].

Modern ICUs generate significant amounts of monitoring data in a continuous stream, providing valuable insights into patients’ prognoses. This has led to a significant focus on leveraging machine learning (ML) and deep learning (DL) approaches to develop more accurate outcome prediction models on the basis of ICU monitoring data [10]. These models demonstrate improved accuracy and reliability by identifying patterns that may not be captured by human analysis or conventional methods, using sophisticated algorithms to evaluate large amounts of time series data [11].

Recent studies have identified key predictive factors for monitoring TBI patients in the ICU [12]. Electrocardiograms (ECGs) are commonly used in ICUs to monitor heart function after injury continuously. Importantly, ECG data have been found useful in predicting mortality risk. Heart rate variability (HRV) is a key measure derived from ECG analysis that quantifies variations in the intervals between successive heartbeats and serves as a measure of autonomic nervous system (ANS) regulation of cardiac activity. Importantly, HRV measures have been established as predictors of early outcomes in TBI patients [13, 14]. Furthermore, ECG abnormalities and patterns are strongly associated with adverse outcomes in TBI patients [15], helping identify patients at high risk of developing autonomic dysfunction, a common complication following TBI [16]. In this context, Lu et al. [17] reported time-based and frequency-based domains HRV features are significant predictors of one-year mortality and functional outcomes in TBI patients. These findings underscore the relevance of HRV in assessing the severity of TBI patients early and aiding in the prognosis. Similarly, Lee et al. [18] demonstrated that the results of the first 24 h of ECG data can be used to determine the severity of a brain injury as well as subsequent patient outcomes, highlighting the importance of early and accurate ECG analysis. Thus, HRV clearly plays a critical role in assessing outcomes and mortality risks in ICU environments, especially when analysed within the crucial early hours following a patient's admission.

This paper introduces a combined HRV feature extraction and hybrid deep learning approach. By capturing the dynamic fluctuations in patients’ HRV, this study develops a novel DL hybrid model integrating a custom weight predictor layer with bidirectional long short-term memory (BiLSTM) recurrent layers. The proposed method is designed to predict in-ICU mortality risk for TBI patients by analysing the first 24 h of ECG data following patient admission. Section 2 reviews the relevant research studies, while Sect. 3 outlines the proposed methodology. Section 4 presents the results, followed by a discussion in Sect. 5. Finally, Sect. 6 summarises the key findings and future research directions.

## Related Works

Existing prediction models utilise a variety of clinical parameters, including the Glasgow Coma Scale (GCS) score, computed tomography (CT) findings, intensive care unit (ICU) admission data, and other biomarkers with machine learning algorithms, to increase prediction accuracy. For example, Shi et al. [12] used a combination of clinical inputs, including hospital volume, Charlson score, length of stay, and demographic data, to predict post-TBI surgery mortality. Their findings demonstrated that an artificial neural network (ANN) outperformed a logistic regression (LR) model, achieving a prediction accuracy of 95.23% compared with that of 82.44% for the LR model. In a retrospective multicentre study [19], EHRs were analysed including emergency medical service and hospital records, surgical and laboratory report data, and communication logs. On the basis of these data, 30-day mortality prediction models were developed, with accuracies reaching 84%. Abujaber et al. [20] subsequently predicted the in-hospital mortality of TBI patients via demographic features, injury characteristics, and CT findings as predictors. The ANN and support vector machine (SVM) algorithms achieved accuracies exceeding 91% and areas under the curve (AUCs) of over 93%.

Daley and colleagues [21] developed several ML-based models to predict severe TBI via neurological and biological data obtained upon admission. Among these models, the random forest (RF) approach achieved a mortality risk prediction accuracy of 82% and an AUC of 90%. Similarly, another study [22] introduced a gradient-boosting classifier (GBM) to estimate risk for trauma patients on the basis of static and dynamic information, achieving an AUC of 92.9%. Recently, Khalili and colleagues [23] explored the use of ML models for short-term and long-term mortality prediction in TBI patients. This study utilised demographic characteristics, laboratory test results, imaging indices, and clinical characteristics with random forest (RF) and decision tree (DT) algorithms. According to their findings, the generalised linear model approach was found to be most effective in predicting a patient’s long-term survival, with an accuracy rate of almost 82%. Conversely, the RF model demonstrated the most effective performance for predicting short-term mortality. Building on recent progress, Ding et al. [24] recently conducted a study and reported an AUC of up to 87.8% by combining EMR and patient monitoring system data to predict in-hospital mortality and neurological outcomes.

Various time series modelling techniques, including RF, light gradient boosting (LightGBM), and LR, have been employed to analyse physiological and clinical information collected within the initial 24 h of an ICU stay for outcome prediction [25, 26]. For example, Palepu et al. [26] derived clinical assessment data, laboratory test results, medication records, and physiological time series data to build a mortality prediction model utilising an Elastic Net linear approach. Their findings indicate that computational evaluation of routinely recorded patient data within the first day of ICU admission effectively predicted ICU outcomes, achieving an AUC of 90.3%. Similarly, another study [25] explored a combination of clinical indicators, such as vital sign data, ICU coma scale scores, pupillary response, intubation status, external ventricular drainage, and comorbidities, to develop mortality prediction models using LR, RF, LightGBM, and XGBoost with various feature combinations. These models achieved performance, with AUC values ranging from 87.7% to 92.1%.

In addition to conventional clinical data, HRV has shown promise as a critical factor for outcome prediction and severity assessment in ICUs. For example, Sykora et al. [27] and Zhang et al. [28] built classification models utilising logistic regression with different feature sets, including HRV-based characteristics. In one study [27], a predictive AUROC of 84.4% was obtained with a combination of classical and autonomic HRV variables. In comparison, Zhang et al. [28] predicted ICU brain injury patient outcome, with an AUROC of 77%. Luo et al. [29] examined the potential of trauma assessment methods and short-term spectral HRV indices as independent predictors of 30-day mortality in trauma patients and achieved an accuracy of 92.4% with a linear logistic regression model. The results of these studies suggest that HRV analysis, particularly within 24 h after admission, can significantly improve outcome prediction and lead to the development of integrated clinical decision support systems and personalised TBI patient care.

A major shortcoming of most existing models is that they depend on static data collected during admission or specific isolated assessments such as GCS scores, laboratory results, or imaging findings. These data points often provide only a limited snapshot of the patient’s evolving condition. Consequently, those approaches are unable to capture critical dynamic changes in physiology and important temporal patterns. Developing predictive models for TBI patients and addressing patients’ temporal variability are also challenging. Time-series approaches often rely on basic summary statistics and thus discard much of the sequential information. Moreover, many predictive models face challenges related to data consistency and availability, which limiting the generalisability and reliability of predictive analytics across diverse ICU settings. Additionally, there is a lack of consensus on the most effective machine learning approaches for outcome prediction. Together, these limitations motivated this work to develop an adaptive, efficient predictive model that is capable of integrating and analysing long-term ECG data.

## Proposed Methodology

**Fig. 1:**
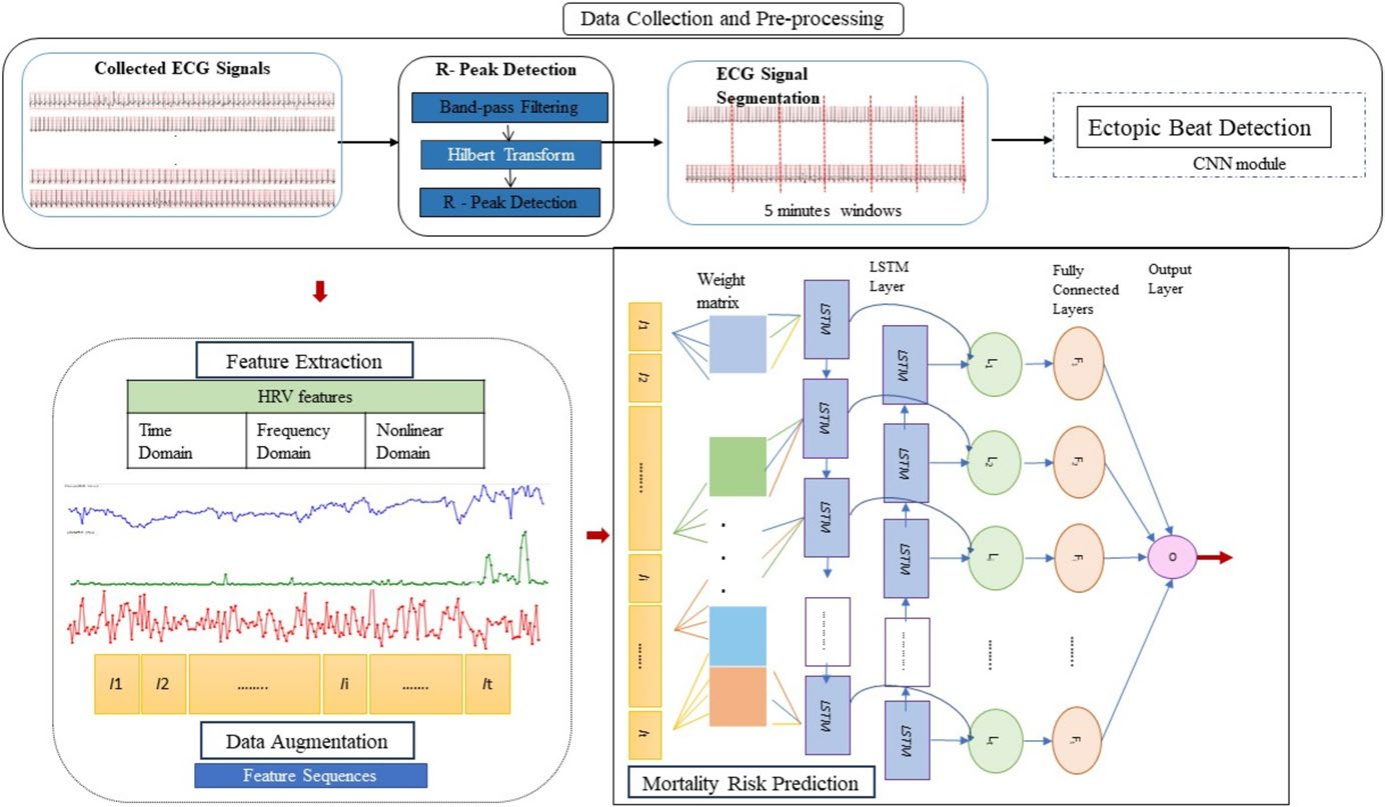
Diagram of our framework. ECG; electrocardiogram, LSTM; long-short term memory, HRV; heart rate variability

In-ICU mortality risk prediction for TBI patients is formulated as a binary classification problem. The methodology consists of four key components: data collection and preprocessing, feature extraction, data augmentation, and mortality risk prediction. Each step is briefly described in the following sections, and Fig. 1 presents a visual representation of the proposed approach. The effectiveness of the proposed model was assessed during the evaluation phase using several cutting-edge ML/DL algorithms. This comparative analysis aims to highlight the strengths and limitations of each approach, and understand the proposed model’s ability to predict ICU mortality risk.

### Data Collection and Preprocessing

This study utilised deidentified data from adult TBI patients (aged > 16 years) who were admitted to the intensive care unit (ICU) at Gold Coast University Hospital (GCUH) in Australia. Data from June 2020 to August 2023 were obtained through Datarwe’s^1^ clinical platform. Patients lacking ECG data were excluded from the study. For additional robustness, we expanded the size of the dataset by extracting data from the Cerebral Haemodynamic Autoregulatory Information System (CHARIS) database,^2^ which contains TBI data from the Robert Wood Johnson Medical Centre of Rutgers University [30]. ECG data were extracted from both datasets for the first 24 h after admission.

The primary outcome of interest was in-ICU mortality during the initial admission, categorised as low risk (survivors) or high risk (nonsurvivors). Discharged ICU patients or those transferred to a ward were considered low risk. Initially, the GCUH data comprised 78 TBI patients. However, 34 patients were subsequently excluded for various reasons, including a lack of ECG recordings, missing long-duration ECG data, ICU discharge within 24 h of admission, or mild admission cases with GCS scores of 14–15. Consequently, combined with data from 13 additional patients from CHARIS, the final dataset consisted of 41 survivors and 16 nonsurvivors.

In the GCUH dataset, raw ECG signals were sampled at 240 Hz. The CHARIS dataset, which originally sampled data at 50 Hz, was upsampled to 240 Hz to standardise the sampling frequency and ensure uniformity across datasets. A high-pass filter and low-pass filter were subsequently applied to eliminate baseline wander and high-frequency noise from the ECG signals. This step enhances ECG signal quality and ensures data integrity. In the next part of the preprocessing procedure, the highest peaks of a heartbeat (R-peaks) were identified using the Hilbert transform method [31]. The ECG signals were subsequently segmented into 5-min intervals to facilitate HRV analysis. A significant challenge in HRV analysis is the presence of ectopic beats, which can introduce noise. Thus, a deep learning model was employed to detect ectopic beats, including ventricular and supraventricular beats, via proposed ectopic beat detection method [32]. The ectopic beats were then filtered from the calculation to ensure the accuracy and reliability of the HRV analysis.

### Feature Extraction

The HRV metrics, both time domain and frequency domain, have been studied and shown as prognostic factors for predicting outcomes in TBI patients in the ICU. Time domain metrics of HRV provide detailed characteristics of autonomic nervous system (ANS) dynamics, and some have been reported being predictive of mortality in critical care settings [33, 34]. Frequency domain metrics capture sympathetic-parasympathetic balance and some have been validated as outcome predictors after TBI [35]. Non-linear measures quantify complex heart-brain interactions that are not visible in linear measures and have shown additional predictive power. Furthermore, studies have proven that HRV metrics are strongly associated with ICU outcome scores at follow-up, demonstrating the relevance of HRV in assessing long-term neurological recovery [36, 37]. A recent review article further highlights HRV as a valuable indicator for assessing the trajectory of recovery following a TBI [38]. According to the predictive power, the predictive value of 24-h recordings is the optimal choice for comprehensive risk stratification [39–41]. Consequently, this study employed a combination of linear and nonlinear HRV as the prognostic factors, given their demonstrated significant potential as clinically relevant variables for monitoring and predicting outcomes in patients with TBI.

**Fig. 2:**
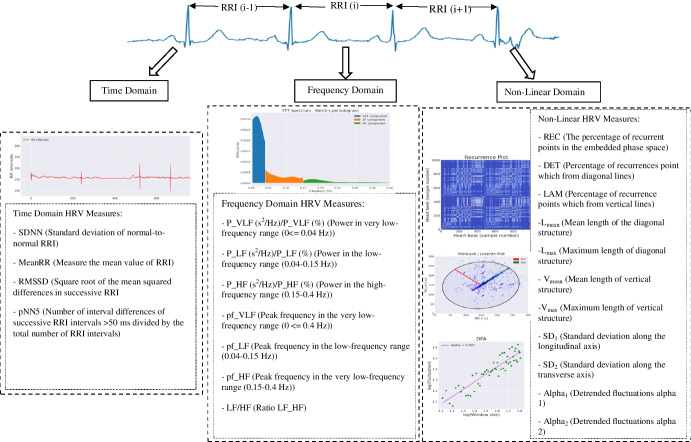
Time domain, frequency domain, and nonlinear domain analysis of heart rate variability. The RRI was obtained by detecting R peaks. Power spectral density (PSD) of the RRI across three frequency bands. Nonlinear HRV were derived based on recurrence quantification analysis (RQA), Poincare plot and detrended fluctuation analysis (DFA)

In this study, we calculated all time domain and frequency domain HRV parameters listed in the literature, on the basis of the identified R peaks for each segment. The RR intervals (RRIs) were calculated, representing the duration between two consecutive R peaks. These parameters provide perspectives on different types of variability (see Fig. 2) [42]. The extracted HRV measures include time domain measures such as the mean RRI (MeanRR), standard deviation of RRI (SDNN), square root of the mean of the squared successive differences between adjacent RR intervals (RMSSD), and percentage of successive RR intervals that differ by more than 50 ms (pNN50). In addition, nine frequency-domain measures were calculated to analyse the spectral components of the RRIs. This included very-low-frequency (VLF), low-frequency (LF), and high-frequency (HF) spectral power and the LF/HF ratio. For nonlinear analysis, seven measures were derived from recurrence quantification analysis (RQA) [43], including the recurrence rate (REC), the percentage of recurrence points forming diagonal lines (DET), the mean length of structures, both diagonal and vertical structures (L_mean_ and V_mean_), and the maximum length of structures of both diagonal and vertical structures (L_max_ and V_max_). In addition, Poincare plot [44] parameters, such as the standard deviation along the longitudinal axis and the transverse axis (SD1 and SD2), and two parameters from detrended fluctuation analysis (DFA) (alpha_1_ and alpha_2_) were calculated to measure short- and long-term fluctuations. The final result consisted of a time series of 288 data points for each measurement and each patient. Missing values were replaced using the k-nearest neighbour (k-NN) imputation strategy. Finally, the data were normalised before being fed into machine learning models.

### Data Augmentation

We employed data augmentation techniques to address the challenge of class imbalance and increase the sample size. Class imbalance occurs when a particular class is significantly underrepresented compared with other classes, leading to biased model performance. In this dataset, only 28.1% of patients did not survive in the ICU. The survival and nonsurvival rates in the training-validation set were 71.2% and 28.8%, respectively. Such disparities increase the risk of the model overfitting to the majority class during training, leading to inadequate performance on unseen data owing to the ability of the model to predict minority cases. Thus, we applied synthetic minority oversampling technique (SMOTE) [45] to both the GCUH and CHARIS datasets to overcome this challenge and increase dataset diversity within each cross-validation fold. SMOTE first flattens each fixed-length HRV time-series into a single-dimensional vector, then employs k-NN clustering in that high-dimensional space to generate new synthetic minority-class samples. The synthetic samples were balanced at a 1:1 ratio between classes to prevent direct duplication. Instead, interpolation was used to create new, unique samples. Padding and masking techniques were implemented to manage various lengths of sequences among subjects. The input sequences were sorted and padded to match the length of the longest batch sequence. The masking process ensures that the network ignores the padding elements. It maintains the integrity of the original sequences. For additional details, please refer to Sect. 2 of the supplementary material.

### Hybrid Network Incorporating a Weight Predictor and a Bidirectional Unit

Hybrid deep learning methods have emerged as highly effective and adaptable methods that combine various advanced techniques to address complex tasks. The proposed model combines a weight predictor and BiLSTM recurrent layers [46]. The architecture consists of an input layer, a custom weight predictor layer, a multiplication operation between inputs and weights, BiLSTM layers, dropout layers, and an output layer, as shown in Fig. 1. The weight predictor serves for weighting HRV features as a feature learning mechanism. It enhances predictive capacity by identifying essential features while suppressing less relevant features, and reducing noise. Simultaneously, the model employs a bidirectional architecture that allows the network to analyse long-term and short-term patterns in the time series data from both past (backwards) and future (forwards) directions. This approach is particularly beneficial for tasks requiring an understanding of pattern changes, such as identifying mortality risk.

#### Weight Prediction Network Layer

Margeloiu et al. [47] proposed a weight prediction network combined with a feature selection method to handle high-dimensional yet small dataset. Similarly, to reduce the number of learnable parameters and to perform feature selection within the network, we designed a custom layer that integrates the weight prediction network. In the input reduction process, the mean values of inputs across the time dimension were computed to reduce their dimensionality (Eq. 1). While the approach in [47] utilised a single dense layer for weight assignment, our weight predictor consisted of two sequential dense layers, each with specific roles. The first layer predicts initial weights via tanh activation, allowing the model to assign positive and negative emphases (Eq. 2). A sigmoid activation function was applied as the second dense layer to ensure that the weights were between 0 and 1 [48] (Eq. 3). To mitigate overfitting, this sparsity was essential for reducing the influence of less informative features. The final computed weights were uniformly applied across all time steps to moderate the input sequence (Eq. 4). Each input was multiplied elementwise by its corresponding weight. Then, the sequence was fed into the LSTM layer for further analysis. By doing so, a weight feature-wise attention mechanism was implemented via this weight predictor rather than adopting a self-attention block, which would introduce multiple large projection matrices, complexity in sequence length, and many extra parameters. Thus, unlike static feature selection methods or heavyweight attention mechanisms, this method enables the importance of features to be determined dynamically during training. Additional details about the mechanism of the weighted predictor are provided in the supplementary Sect. 3.

1 $$r=\frac{1}{T}\sum_{ t=1}^{ T}{x}_{t}$$

2 $${w}_{pred = \text{tanh} ({ W}_{pred }r+ {b}_{pred} )}$$

3 $${w}_{sparse}= \sigma ({W}_{sparse} {w}_{pred}+ {b}_{sparse})$$

4 $${X}_{weighted}=X.{w}_{sparse}$$

where, ${x}_{t}$ is the value of the feature at time step $t$, $T$ is the total number of time steps, $r$ denotes the single mean value for the feature, ${W}_{pred}$, ${b}_{pred}$ are the weights and bias of the dense layer, ${W}_{sparse}$, ${b}_{sparse}$ are weights and bias of the sparsity layer, $\sigma$ denotes the sigmoid activation function, ${X}_{weighted}$ is the sequence weighted by the learned importance of each feature, $X$ is the original input sequence.

#### Bidirectional LSTM Layer

This layer processes the weighted input and consists of an LSTM unit in a bidirectional wrapper. It simultaneously processes the input sequence in both forwards and backwards directions, allowing the model to capture temporal dependencies from past and future time steps. This approach is more efficient than a one-way LSTM model is, as both preceding and subsequent steps inform the output at each time step [49]. The memory unit includes the input gate (${\Gamma }_{i})$, forget gate (${\Gamma }_{f})$, and output gate (${\Gamma }_{o})$, which control the information that is read, stored in the internal memory, or passed [40].

**Fig. 3:**
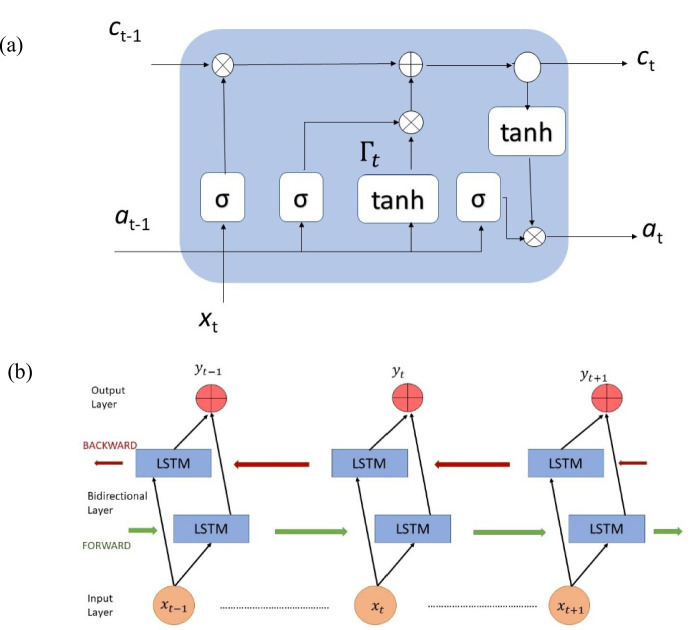
(**a**) LSTM structure. (**b**) BiLSTM structure

Figure 3 shows a typical LSTM cell with gates and a BiLSTM cell, adopted from [50]. At time t, the input is $${x}_{t}$$, the previous output of the hidden layer is $${a}_{t-1}$$, and the current output is $${a}_{t}$$. Let $$\overrightarrow{{a}_{t}}$$ represent the forwards LSTM’s hidden state and $$\overleftarrow{{a}_{t}}$$ represent the backwards LSTM’s hidden state. $$\widetilde{{c}_{t}}$$ and $${c}_{t}$$ are the input and output states, respectively. The previous state is $${c}_{t-1}$$. We compute the three gate states, the cell input state, the cell output ($${c}_{t})$$ and the hidden layer output ($${a}_{t})$$ using the following equations in order.

5 $${\Gamma }_{i}=\sigma ({W}_{i}[{a}_{t-1 },{x}_{t}]+{b}_{i})$$

6 $${\Gamma }_{f}=\sigma ({W}_{f}[{a}_{t-1 },{x}_{t}]+{b}_{f})$$

7 $${\Gamma }_{o}=\sigma \left({W}_{o}[{a}_{t-1 },{x}_{t}]+{b}_{o}\right)$$

8 $$\widetilde{{c}_{t}}=\text{tanh}({W}_{c}[{a}_{t-1 },{x}_{t}]+{b}_{c})$$

9 $${c}_{t}={\Gamma }_{i}*\widetilde{{c}_{t}}+{\Gamma }_{f}* {c}_{t-1}$$

10 $${a}_{t}={\Gamma }_{o}*\text{tanh}\left({c}_{t}\right)$$

where ${b}_{i}, {b}_{f}, {b}_{o} and {b}_{c}$ are bias vectors and ${W}_{i}, {W}_{f}, {W}_{o} and {W}_{c}$ are weight matrices to the input, forget, output, and cell state, respectively. The sigmoid function is represented by $\sigma$, and the hyperbolic tangent function is represented by $\text{tanh}$.

### Experimental Preparation

#### Model Training and Hyperparameter Tuning

The dataset was divided into two sets: the training dataset, which comprised 80% of the total data, was used for training and validation, and the remaining 20% was used for model performance testing. The performance metrics were derived from both the validation and test datasets. During the training phase, a fivefold cross-validation strategy was conducted on the training-validation dataset to refine model selection, adjust hyperparameters, and determine the model architecture. To identify the optimal hyperparameters, different configurations were systematically tested, including the number of LSTM neurons (16, 32, 64, 128, and 256), the number of hidden layers (1 or 2), and the dropout rate (0, 0.25, and 0.3). The model was trained over 100 epochs using the adaptive moment estimation (Adam) optimiser [51] with a batch size of 32 and a fixed learning rate of 0.001. The ReLU activation function was used to introduce nonlinearity and enhance pattern recognition capabilities. To address the challenge of overfitting, dropout layers were strategically included in the network architecture [52]. The output layer applies the softmax function to normalise the output probabilities, and binary cross-entropy serves as the loss function to measure the error between the predicted and actual outputs. Following the training process, the best model was applied to the test set to evaluate the final test metrics. We trained the model using the multiclass cross-entropy loss function and conducted all the experiments using the Python programming language with TensorFlow on AWS Amazon’s^3^ cloud platform.

#### Model Evaluation Metrics

The sensitivity, specificity, positive predictive value (PPV), accuracy, and F1 score were used to evaluate the model performance. These metrics were calculated via standard expressions for TP, FP, TN, and FN, which refer to “true positives,” “false positives,” “true negatives,” and “false-negatives,”, respectively, as represented in Eqs. (11) to (15). Additionally, we used the area under the receiver operating characteristic curve (AUROC) and the area under the precision‒recall curve (AUPRC) to evaluate the model. Confidence intervals were computed via a bootstrap procedure with 10000 unstratified resamples. Weighted averages of these performance metrics were calculated to provide an overall assessment.

11 $$Sensitivity= \frac{TP}{TP+FN}$$

12 $$Specificity= \frac{TN}{TN+FP}$$

13 $$PPV=\frac{TP}{TP+FP}$$

14 $$Accuracy= \frac{TP+TN}{TP+FP+TN+FN}$$

15 $$F1= \frac{2*Precision*Recall}{Precision+Recall}$$

#### Feature Importance Analysis

A feature importance analysis was performed in order to identify the key critical features that contribute to the prediction of mortality. The permutation feature importance technique was employed to evaluate the contribution of each HRV feature. This method calculates the mean absolute error (MAE) via the binary cross-entropy as the loss function to compare the model’s predictions with the actual results. Each HRV feature in the dataset was systematically permuted by shuffled its values at random. An increase in the MAE upon permuting a particular feature indicates its relative importance to the predictive accuracy of the model. The MAEs of each permuted dataset were compared with the baseline MAEs, to quantify the contributions of individual HRV features to the model performance. This analysis highlighted the most influential features, offering insights into the underlying physiological dynamics captured by the model.

## Results

Using statistical methods, we examined the differences in HRV characteristics between TBI patients’ survivors and nonsurvivors. The results are detailed in Sect. 4 of the supplementary material, and the statistical results are presented in Table S1 and Figure S1. The results revealed significant differences in several HRV characteristics between the two groups. For example, patients who survived presented significantly higher SDNN, MeanRR, and pNN50 values than nonsurvivors did. Significant differences were also observed in SD1 and SD2, which are measures of short-term and long-term HRV variability, respectively. To evaluate the effectiveness of the proposed model, we optimised the hyperparameters through a fivefold cross-validation process via a grid search technique. The model was then trained on the training dataset, and its performance was assessed on the validation dataset. The optimal model configuration included a custom weight predictor, a 16-unit BiLSTM layer, and an L2 regulariser to prevent overfitting. With this configuration, the model achieved a mean validation AUROC of 0.995 and an AUPRC of 0.998. Figure S2 in the supplementary material shows the model validation performances across different BiLSTM configurations, with kernel sizes of 16, 32, 64, and 128. The dropout layer was set to 0.3. The dense layer included 20 neurons with ReLU as the activation function. Table 1 presents the validation results, including sensitivity, specificity, PPV, accuracy, AUROC, and AUPRC. Figure S3 in the supplementary material displays dynamic graphs of accuracy, loss, and AUROC during training and internal validation. They highlight the gradual convergence of training and validation errors, indicating that the proposed weight-BiLSTM model does not exhibit overfitting. The training and validation accuracies started at 0% and reached 85% within six epochs, while the loss gradually decreased to 0.01. Furthermore, the AUROC curve, plotted against the true positive rate and false-positive rate (see Figure S3 in the supplementary material), confirmed the superior classification efficiency of the proposed model.

**Table 1:** Comparison of the average fivefold cross-validation performance among numerous existing machine learning/deep learning methods

Model	Sensitivity (95%CI)	Specificity (95%CI)	PPV (95%CI)	Accuracy (95%CI)	AUROC (95%CI)	AUPRC (95%CI)
LightGBM	0.937 (0.846–1.000)	0.925 (0.823–1.000)	0.957 (0.891–1.000)	0.918 (0.869–1.000)	0.969 (0.923–1.000)	0.969 (0.921–1.000)
RF	0.717 (0.333–1.000)	0.953 (0.826–1.000)	0.891 (0.765–1.000)	0.837 (0.615–1.000)	0.902 (0.864–1.000)	0.893 (0.796–1.000)
XGBoost	0.757 (0.498–1.000)	0.663 (0.578–0.833)	0.707 (0.571–1.000)	0.711 (0.615–1.000)	0.904 (0.583–1.000)	0.929 (0.719–1.000)
LR	0.609 (0.25–1.000)	0.964 (0.507–1.000)	0.944 (0.427–1.000)	0.77 (0.5–1.000)	0.915 (0.625–1.000)	0.931 (0.655–1.000)
RNN	0.969 (0.897–1.000)	0.471 (0.181–0.75)	0.818 (0.694–0.925)	0.825 (0.711–0.933)	0.832 (0.673–0.954)	0.925 (0.839–0.984)
LSTM	0.752 (0.594–0.882)	0.614 (0.333–0.857)	0.826 (0.679–0.94)	0.715 (0.578–0.822)	0.777 (0.611–0.916)	0.891 (0.78–0.973)
BiLSTM	0.781 (0.66–0.912)	0.921 (0.75–1.000)	0.961 (0.87–1.000)	0.822 (0.711–0.911)	0.866 (0.714–0.974)	0.928 (0.807–0.993)
BiGRU	0.967 (0.9–1.000)	0.921 (0.737–1.000)	0.968 (0.897–1.000)	0.914 (0.889–1.000)	0.985 (0.948–1.000)	0.994 (0.978–1.000)
weight-BiLSTM	0.937 (0.846–1.000)	0.922 (0.727–1.000)	0.967 (0.893–1.000)	0.933 (0.844–1.000)	0.995 (0.978–1.000)	0.998 (0.99–1.000)

Additional experiments were conducted to (1) confirm the effects of different training data durations on model performance and (2) compare the model’s performance against that of cutting-edge machine learning and deep learning techniques to evaluate the proposed weight-BiLSTM hybrid model.

### Effect of Training Data Duration on Model Performance

**Fig. 4:**
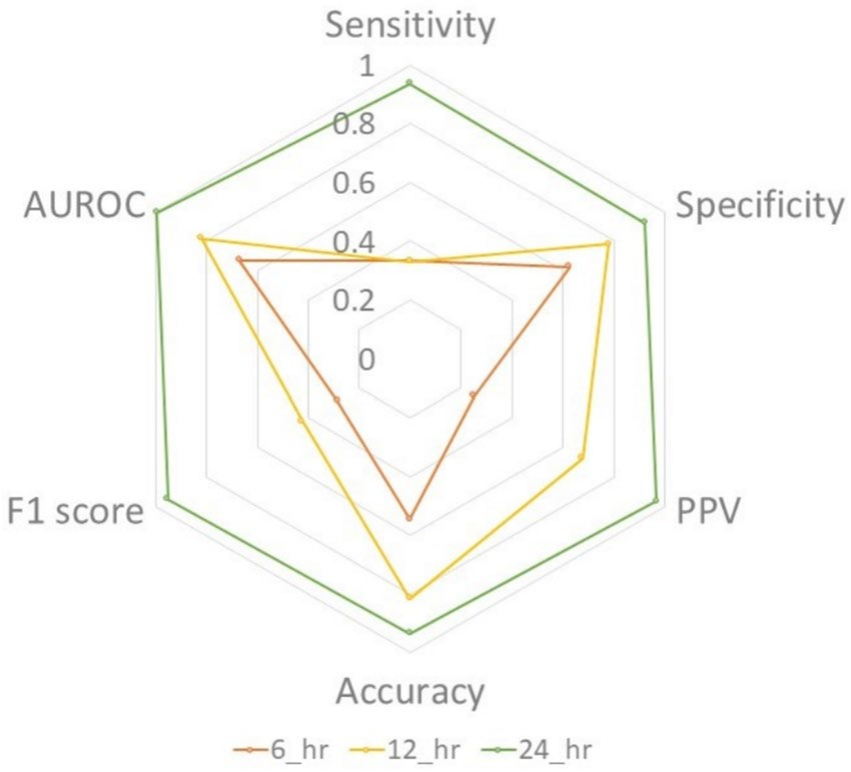
Discrimination validation performance of mortality risk prediction by patient monitoring time

We utilised initial data from 6, 12, and 24 h for mortality risk classification to confirm the effect of training data length on the predictive performance of the proposed model. The model trained with the first 6 h of data achieved a classification accuracy of 0.545 and an AUROC of 0.67. When the training period was extended to 12 h, the accuracy was significantly increased to 0.814 and the AUROC was increased to 0.82. The most notable improvement was observed when the model was trained using the first 24 h of data, achieving an accuracy of 0.933 and an AUROC of 0.967. These results were reflected across all the performance metrics, including sensitivity, which increased from 0.331 with 6 h of data to 0.937 with 24 h of data. Notably, despite incorporating data augmentation, the model demonstrated remarkable stability without signs of overfitting. These results show that the performance of the proposed model achieves peak performance when trained using the first 24 h of data (see Fig. 4). This finding underscores the reliability and consistency of using extended HRV measures for the first 24 h of ECG data in predicting mortality risk in clinical settings.

### Comparison of the Performance of the Proposed Model with that of State-of-the-Art Machine/Deep Learning Techniques

We compared the mortality risk prediction efficiency of the proposed hybrid model against that of eight existing machine learning and deep learning methods. The benchmark models included the light gradient boosting model, random forest, extreme gradient boosting (XGBoost), logistic regression, recurrent neural network (RNN), long-short term memory, bidirectional long short-term memory, and bidirectional gated recurrent unit (BiGRU). The performance analysis of these models in mortality risk prediction is shown in Table 1. These models were all evaluated on the same training and testing datasets. Computed HRV features for each epoch flattened them into a feature vector per patient, yielding a format compatible with tabular algorithms (LightGBM, RF, XGBoost, LR). We trained deep learning models with the following numbers of parameters: RNN with 22,569 parameters, LSTM with 24,489 parameters, BiLSTM with 16,169 parameters, BiGRU with 12,841 parameters, and weight-BiLSTM with 7,357 parameters, as detailed in Table S2 of the supplementary material. The hyperparameter optimization and cross-validation results for the best configurations are also shown in Table S2 of the supplementary material.

Baseline machine/deep learning models and proposed model using fivefold stratified cross-validation on our training cohort are shown in Table 1, and report means ± 95% confidence intervals (CIs) for performance metrics. The proposed weight-BiLSTM model significantly outperformed all the other methods in terms of classification accuracy, AUROC, and AUPRC (Table 1). Specifically, the results suggest that the proposed weight-BiLSTM model achieves the highest classification accuracy of 0.933 (95% CI 0.844–1.000) and the highest AUROC of 0.995 (0.978–1.000), with a sensitivity of 0.937 (0.846–1.000) and a PPV of 0.967 (0.893–1.000). Among conventional classifiers, the LightGBM model achieved an accuracy of 0.918 (95% CI 0.869–1.000), AUROC 0.969 (0.923–1.000), specificity of 0.925 (0.823–1.000), and a sensitivity of 0.937 (0.846–1.000). In contrast, RF and XGBoost showed moderate performance, with RF achieving a specificity of 0.953 (95% CI 0.826–1.000) and XGBoost achieving a specificity of 0.663 (0.578–0.833). LR achieved a specificity and PPV of 0.964 (0.507–1.000) and 0.944 (0.427–1.000), respectively. However, its sensitivity was low at 0.609 (0.25–1.000), indicating a tendency to misclassify low-risk cases. Among the deep learning approaches, the RNN exhibited a high sensitivity of 0.969 (95% CI 0.897–1.000) but a low specificity of 0.471 (0.181–0.75). The LSTM model performed classification with a sensitivity of 0.752 (0.594–0.882), a specificity of 0.614 (0.333–0.857), and a PPV of 0.826 (0.679–0.94). However, the BiLSTM and BiGRU models highlight the strength of bidirectional architectures, with the BiGRU model achieving an almost perfect AUROC of 0.985 (0.948–1.000) and an AUPRC of 0.994 (0.978–1.000). Leave-one-out cross-validation (LOOCV) was performed on the full dataset to address the concern about the patient-level bias. Table 2 summarises the LOOCV results with 95% CIs. The weight-BiLSTM again achieved the highest discrimination, accuracy of 0.947 (0.895–0.987), AUROC of 0.996 (0.986–1.000), AUPRC of 0.996 (0.985–1.000). Also, it maintained excellent sensitivity of 0.92 (0.821–1.000) and specificity of 0.973 (0.915–1.000). LightGBM and XGBoost mirrored each other closely under LOOCV, with AUROCs around 0.875 and accuracies around 0.843, while simpler models like LR dropped further (AUROC 0.738 [0.593–0.866], accuracy 0.685 [0.561–0.807]). The consistency between fivefold CV and LOOCV for our proposed model demonstrates its robustness to inter-patient variability.

**Table 2:** Comparison of the LOOCV performance among numerous existing machine learning/deep learning methods

Model	Sensitivity (95%CI)	Specificity (95%CI)	PPV (95%CI)	Accuracy (95%CI)	AUROC (95%CI)	AUPRC (95%CI)
LightGBM	0.902 (0.806–0.977)	0.69 (0.455–0.9)	0.882 (0.78–0.975)	0.843 (0.737–0.93)	0.874 (0.766–0.972)	0.948 (0.884–0.991)
RF	0.928 (0.837–1.000)	0.312 (0.105–0.533)	0.777 (0.667–0.887)	0.756 (0.649–0.86)	0.823 (0.7–0.929)	0.928 (0.855–0.978)
XGBoost	0.902 (0.795–0.977)	0.693 (0.438–0.9)	0.883 (0.78–0.972)	0.843 (0.737–0.93)	0.875 (0.762–0.974)	0.949 (0.884–0.922)
LR	0.685 (0.538–0.824)	0.684 (0.417–0.909)	0.849 (0.72–0.964)	0.685 (0.561–0.807)	0.738 (0.593–0.866)	0.886 (0.791–0.961)
RNN	0.808 (0.682–0.921)	0.622 (0.4–0.857)	0.844 (0.721–0.949)	0.755 (0.649–0.86)	0.85 (0.729–0.939)	0.942 (0.882–0.979)
LSTM	0.829 (0.714–0.932)	0.689 (0.428–0.917)	0.873 (0.756–0.974)	0.79 (0.684–0.895)	0.892 (0.785–0.963)	0.958 (0.907–0.989)
BiLSTM	0.826 (0.7–0.935)	0.558 (0.308–0.812)	0.826 (0.703–0.939)	0.751 (0.632–0.86)	0.9 (0.806–0.968)	0.964 (0.925–0.990)
BiGRU	0.753 (0.619–0.875)	0.556 (0.3–0.786)	0.814 (0.676–0.923)	0.698 (0.579–0.807)	0.810 (0.679–0.915)	0.928 (0.866–0.972)
weight-BiLSTM	0.92 (0.821–1.000)	0.973 (0.915–1.000)	0.967 (0.893–1.000)	0.947 (0.895–0.987)	0.996 (0.986–1.000)	0.996 (0.985–1.000)

**Fig. 5:**
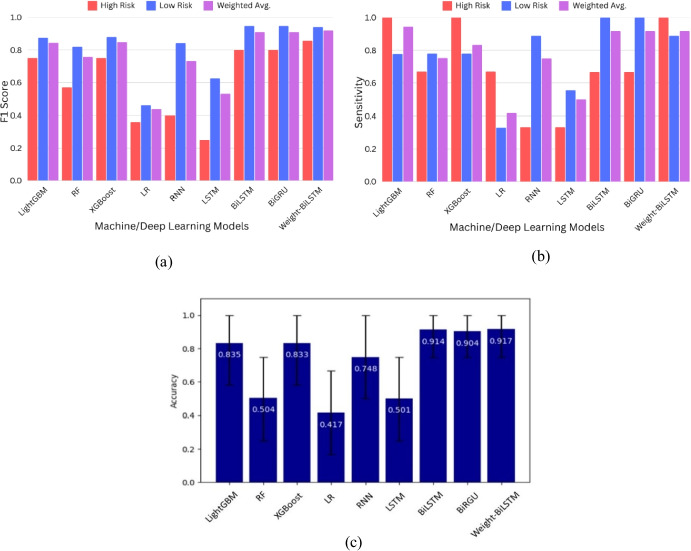
Comparison of the proposed model performance with that of state-of-art methods on the test dataset: (**a**) F1 score, (**b**) sensitivity, and (**c**) overall accuracy

For the independent testing phase, the performance of different prediction methods was analysed, as shown in Fig. 5. The RNN yielded an F1 score of 0.732, an accuracy of 0.748 (95% CI 0.6–0.866), and an AUROC of 0.767 (0.641–0.926). The BiLSTM model outperformed the RNN, with a sensitivity of 0.917 (0.75–1.000), an accuracy of 0.914 (0.75–1.000). Similarly, the BiGRU demonstrated an F1 score of 0.91 and a specificity of 0.75 (0.55–0.952). Among the other ML models, the LightGBM achieved a weighted average sensitivity of 0.944 and an F1 score of 0.844, whereas the RF and XGBoost models achieved weighted average sensitivity values of 0.753 and 0.833, respectively, with corresponding specificity values of 0.611 (0–1.000) and 0.945 (0.5–1.000). Notably, the weight-BiLSTM model achieved a sensitivity of 0.917 (0.898–1.000) and a specificity of 0.972 (0.86–1.000). For high-risk classification, the specificity reached 0.889, whereas for low-risk classification, the specificity was 1.0 (Fig. 5 (a)). The F1 score for high-risk classification was 0.857, whereas for low-risk classification, an F1 score of 0.941 was achieved. The accuracy reached 0.917 (0.75–1.000), whereas the AUROC was 0.926 (0.76–1.000) and the AUPRC was 1 (see Table S3 in the supplementary material). These results indicate that the proposed model achieves better classification performance than the other models do, even when tested on unseen datasets (Fig. 5 and Table S3 in supplementary material).

To further compare and assess the proposed model in capturing the temporal dynamics of HRV against purely convolutional or hybrid architectures, we conducted LOOCV on three additional deep baseline models, including a CNN-LSTM, a VGG-style-1D network, and a ResNet-1D, all trained for up to 200 epochs with early stopping (patience = 5). Table 3 reports their performances alongside our proposed weight-BiLSTM model. As shown, the proposed model used fewer training parameters and layers and exhibits lower average training loss. According to the overall performance in Accuracy, AUROC, AUPRC, and loss, we can infer that the proposed weight-BiLSTM model outperforms the other baseline deep learning models.

**Table 3:** Training performance (LOOCV) comparison between the proposed model and conventional deep learning models

Model	Train parameters	Layers	Accuracy (95%CI)	AUROC (95% CI)	AUPRC (95% CI)	Loss
CNN-LSTM	71,233	9	0.842 (0.737–0.93)	0.962 (0.903–0.995)	0.987 (0.963–0.998)	0.5618
ResNet-1D	36,609	16	0.789 (0.684–0.895)	0.942 (0.874–0.986)	0.989 (0.965–0.999)	0.3434
VGG-style-1D	2,551,617	19	0.938 (0.719–0.930)	0.87 (0.761–0.954)	0.948 (0.887–0.983)	0.4452
weight-BiLSTM	7,357	7	0.947 (0.895–0.987)	0.996 (0.986–1.000)	0.996 (0.985–1.000)	0.147

### Feature Importance Analysis

The weight predictor layer in the proposed model plays a pivotal role in determining the most predictive features early in the process. The output from this layer was used to moderate the input features before being fed into a BiLSTM for processing. The analysis revealed that features such as alpha1, P_VLF (%), LF_HF ratio, Vmax (bts), and alpha2 were assigned high average weights for all 288 epochs (see Figure S4 in the supplementary document). The adaptability of the weight predictor layer enhances input handling, leading to improved predictive accuracy. This improvement is demonstrated by high validation accuracy and high AUROC scores, as shown in Figure S3 of the supplementary material.

**Fig. 6:**
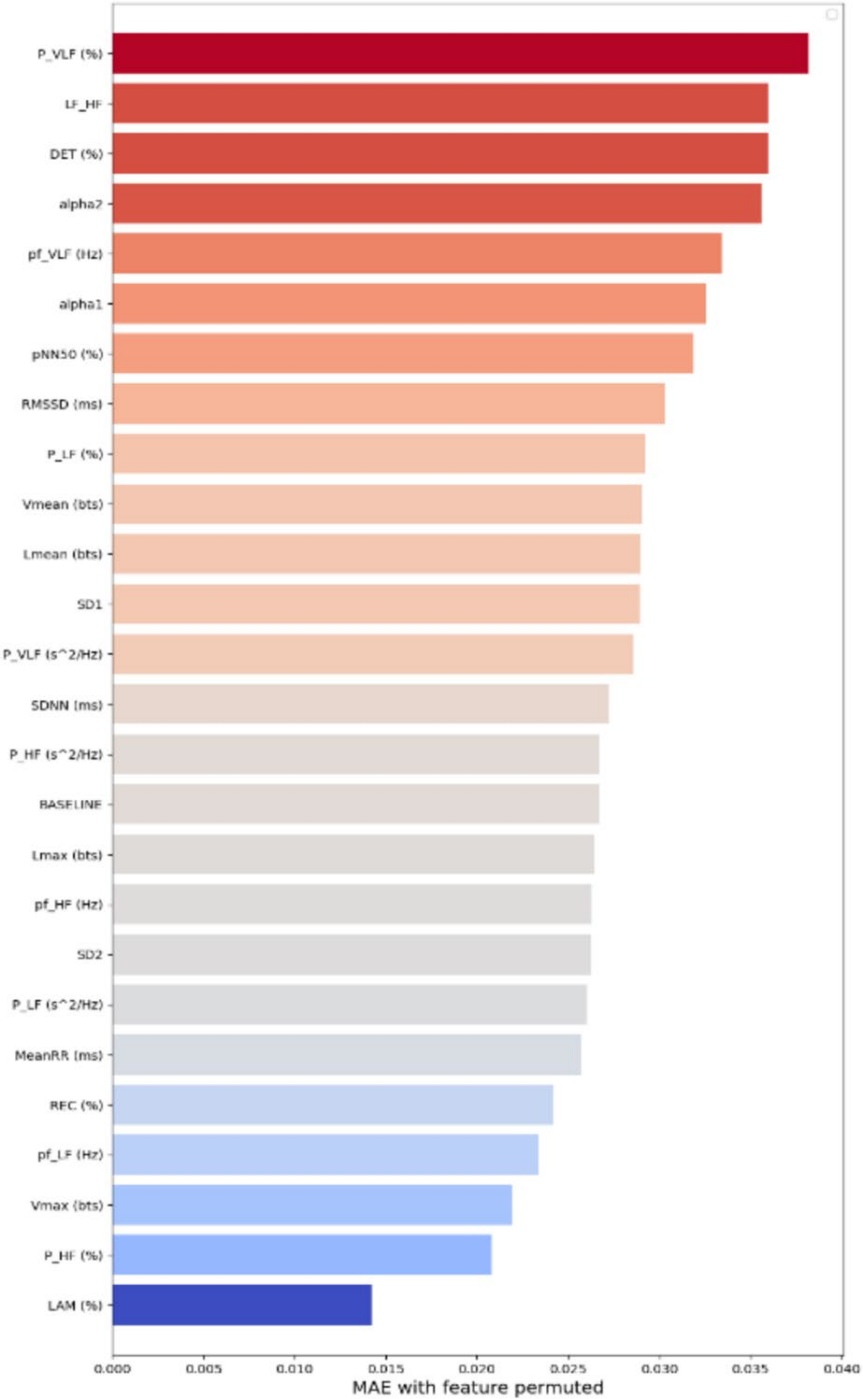
Impact of features on the performance of weight-BiLSTM

The most crucial HRV features identified in the proposed in-ICU mortality prediction model include P_VLF (%), LF_HF, DET (%), alpha2, pf_VLF (Hz), alpha1, and pNN50 (%), as determined using the permutation feature importance approach. The MAE for each feature compared with that for the baseline is presented in Fig. 6. The high importance of these factors could be due to their strong physiological relevance to outcomes in TBI patients. The most influential feature affecting model performance was the very low-frequency range (P_VLF (%)), followed closely by the low-frequency–high-frequency ratio (LF_HF). The percentage of recurrence points that originate from the diagonal lines in the RR interval plot (DET) and detrended fluctuations in the RR interval (alpha1 and alpha2), which are nonlinear domain features, and changes in the percentage of successive RRI intervals from the total number of RRI intervals (pNN50%), which is a time domain analysis feature, all play a significant role in the model’s performance. These features capture variations in heart rhythm associated with autonomic dysfunction. Furthermore, Figure S5 in the supplementary document presents a heatmap illustrating the time-resolved importance of features in the temporal model’s attention. For nearly all features, the most important peaks occur between epochs 20–50, indicating that HRV dynamics within roughly the first 8–10 h offer an especially strong prognostic signal.

## Discussion

The early identification of in-ICU mortality risk among patients with traumatic brain injury is highly important. Early predictions enable clinicians to optimise ICU resource management, initiate timely diagnostics, guide appropriate interventions, and improve patient outcomes in real-world clinical settings. Recognizing this critical need, we developed and validated a simple yet robust deep learning model that uses heart rate variability measures to predict in-ICU mortality risk. Best of our knowledge, this study is the first application of deep learning techniques to predict mortality risk in TBI patients via HRV features alone. This approach enhances predictive accuracy and offers valuable prediction that is helpful for clinical decision-making.

This study highlights the model’s ability to predict in-ICU mortality risk based solely ECG-derived HRV data. The usability and interpretability of the proposed DL-based model are enhanced by its reliance on HRV measures alone. Thus, this approach is easy to use and adaptable to other health care settings if continuous ECG monitoring is standard practice. Streamlined data utilisation not only simplifies clinical workflows but also allows the model to be readily adopted without requiring additional data collection. Thus, this approach overcomes the limitations of conventional prediction models that are based on large amounts of EMR data. Moreover, the robustness of our methodology is demonstrated by consistently high validation accuracy and AUC scores, underscoring the predictive validity of the weighted HRV features.

The model was trained separately using data collected at 6, 12, and 24 h after initial ICU admission. The findings demonstrated the potential of 24-h time series HRV measures for reliable ICU mortality risk assessment. Although HRV measures have been used in previous studies to develop predictive models across various medical contexts, they have frequently depended on conventional statistical methods. The main limitation of these methods is that they assume a linearity between predictors and outcomes. In contrast, the proposed DL-based model effectively captures complex and nonlinear relationships between predictors and outcomes, allowing the use of multiple HRV measures as sequential data. In this study, 25 HRV measures were used as input features, significantly enhancing the accuracy and robustness of the model’s predictions. Among these, the most essential HRV features identified through feature-importance analysis were P_VLF (%), LF_HF, DET (%), alpha2, pf_VLF (Hz), alpha1, and pNN50 (%). Frequency-domain HRV features such as P_VLF and LF_HF provide insights into sympathetic nervous system activity. P_VLF is particularly relevant among these features, as previous literature suggests its link to autonomic nervous system function and potentially to other slow-acting physiological processes. Similarly, the LF_HF ratio signifies the balance between sympathetic and parasympathetic activities, serving as a marker of ANS regulation. These results were consistent with the results of previous meta-analyses [53] and prospective observational studies [54], demonstrating the strong predictive ability of LF_HF for separating survivors from nonsurvivors.

**Fig. 7:**
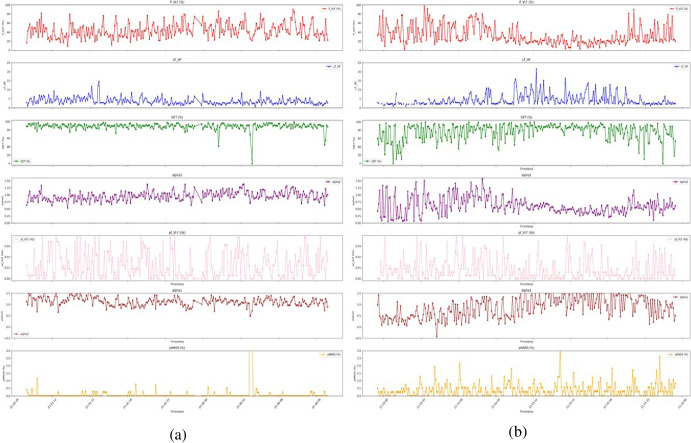
Comparison of the seven most important HRV measures over the first 24 h for a patient who survived and a patient who did not survive from GCUH. (**a**) the figure displays data from that who survived, (**b**) shows data from patient who did not survive

Additionally, time-domain features such as pNN50 provide insights into the overall variability and distribution of RR intervals. This measure is frequently used to evaluate HRV in relation to parasympathetic activity, including emotional stress and cardiovascular health. Figure 7 shows variations in these key HRV measures over the first 24 h for two patients from the GCUH dataset. There was one survivor and one who nonsurvivor. Notably, the LF_HF ratio consistently presented higher values and fluctuations in nonsurvivor than in survivor. These observations align with the findings of Su et al. [15], indicating that the degree of brain-stem damage correlates with increased sympathetic activity, decreased parasympathetic drive, and increased LF_HF ratio.

This study utilised two databases, which enhances the representativeness and generalizability of the results across various patient populations and health care systems. However, there are several limitations to address. First, the cohort size used was a notable constraint, which is a common challenge in AI-based health studies. Second, the proposed model is currently designed to predict patient in-ICU mortality risk but does not provide insights into the timing of these predicted events or access long-term functional outcomes. Third, the model overlooks the impact of treatment on mortality risk, as it focuses solely on HRV predictors without treatment-related variables. Fourth, the model requires 24 h of HRV data for prediction, excluding patients who experience mortality or critical events within the first 24 h of ICU admission. As such, the model is limited in its ability to predict outcomes for patients who survived/did not survive during the initial 24-h period. Finally, the study lacked an external validation set from another independent clinical environment, and the proposed model was compared only against conventional ML/DL methods, as no previous works have utilised HRV indicators alone for mortality prediction. Further studies should aim to validate the results via larger datasets to increase the model’s generalizability and the reliability of its predictions. Moving forward, developing a real-time monitoring model that considers the timing of mortality risk would provide deeper insights into patient trajectories and support improvements in the long-term outcomes of TBI patients in ICU settings.

## Conclusion

In this study, HRV analysis was used to develop a DL-based algorithm for predicting in-ICU mortality risk in patients with traumatic brain injuries. We propose that the weight-BiLSTM model effectively captures and handles the complex temporal dynamics inherent in time series features. It relies solely on ECG data and can be easily implemented in clinical settings. The model was interpretable and highlighted predictive HRV features that warrant further investigation. While our study focused primarily on TBI patients, it has the potential to be expanded to evaluate other types of critical care in the future. The insights gained from this study are expected to aid in developing a real-time monitoring system for the intensive care unit, enhancing patient management and outcomes.

## Supplementary Information

Below is the link to the electronic supplementary material.

Supplementary file1 (DOCX 1768 KB)

## Data Availability

The de-identified GCUH dataset utilised for this study is available upon reasonable request from Datarwe Pty Ltd. Access will be granted to researchers who meet the criteria for confidential data approval as stipulated by the governing protocol. All original code has been deposited at Github at https://github.com/HasiUdayangi/TBI-HRV-Prediction.git and is publicly available as of the date of publication.

## Footnotes

1 https://datarwe.com

2 https://physionet.org/content/charisdb/1.0.0/

3 https://aws.amazon.com/

## Data Availability

The de-identified GCUH dataset utilised for this study is available upon reasonable request from Datarwe Pty Ltd. Access will be granted to researchers who meet the criteria for confidential data approval as stipulated by the governing protocol.
