# Supplementary material for: A Hybrid Bidirectional Deep Learning Model Using HRV for Prediction of ICU Mortality Risk in TBI Patients
Source: J Healthc Inform Res. 2025 Jul 30;9(4):629–55. doi: 10.1007/s41666-025-00209-5 (PMC12602819; doi:10.1007/s41666-025-00209-5)
Supplement: Supplementary file 1 — Supplementary file1 (DOCX 1768 KB) [file 41666_2025_209_MOESM1_ESM.docx]

Supplementary Material: A Hybrid Bidirectional Deep Learning Model Using HRV for Prediction of ICU Mortality Risk in TBI Patients

Hasitha Kuruwita A.^1^, Shu Kay Ng^2^, Alan Wee-Chung Liew^3^, Kelvin Ross^4^, Brent Richards^5^, Kuldeep Kumar^6^, Luke Haseler^7^ and Ping Zhang^1*^

^1^ School of Medicine and Dentistry, Griffith University, Gold Coast, Australia

^2^ School of Medicine and Dentistry, Griffith University, Nathan, Australia

^3^ School of ICT, Griffith University, Gold Coast, Australia

^4^ Datarwe, Gold Coast, Australia

^5^ IntelliHQ, Gold Coast, Australia

^6^ Bond University, Gold Coast, Australia

^7^ Curtin School of Allied Health, Curtin University, Perth, Australia

^*^Correspondence: [p.zhang@griffith.edu.au](mailto:p.zhang@griffith.edu.au)

Table of content Page

Table of content 2

Novelty in our methodology 3

Handling variable sequence lengths in networks 3

Mechanism of weight predictor 3

Table S1: Statistical analysis of heart rate variability (HRV) features between survivors and nonsurvivors. 5

Table S2: Comparison of best cross validation performance between proposed model and other approaches. 6

Table S3: Comparison of performance with state-of-the-art methods on the test datasets. 7

Figure S1: HRV feature changes over 24 hours for survivors and nonsurvivors. 8

Figure S2: (a) Performance of weight-BiLSTM with different kernel size in the validation dataset. (b) Performance of Weight-BiLSTM with different kernel size in the LOOCV. 9

Figure S3: Dynamic graph of proposed model (weight-BiLSTM) (accuracy, loss and AUROC analysis). 10

Figure S4: Mean attention weight per HRV features 11

Figure S5: Time-resolved HRV features importance. 11

1. Novelty in our methodology:

The key points of novelty in our methodology are: (1) application of bidirectional long short-term memory (BiLSTM) Networks to longitudinal heart rate variability (HRV) features, enabling to learn temporal changes and extract important information relating to mortality; (2) use weight predictor as a feature selection mechanism that is integrated within the network, thereby enhancing model robustness and reducing overfitting.

1. Handling variable sequence lengths in networks:

Data collected from patients can vary in sequence length due to missing data. To standardize input lengths without losing the integrity of the time-series data, we employ padding and masking techniques.

Sequence standardization through padding.

Each patient’s data in initially sorted by sequence length, with the longest sequences positioned first. This arrangement aids in efficient batch processing by the layers. All other sequences in the batch are then extended to match the length of the longest sequence, which in this study is set to 288-time steps, corresponding to 24 hours of data captured in 5-minutes segments. Padding involves appending zeros to the end of shorter sequences to achieve this uniformity.

Preservation of data integrity through masking.

Simply padding sequences can lead to misleading interpretations and erroneous processing by the BiLSTM network, as the model might treat padding as valid data. To prevent this, a masking layer is applied immediately after the input layer. This layer creates a mask that flags the padded values. This approach allows the model to maintain the temporal integrity of shorter sequences without interference from padding. By implementing these techniques, the model effectively handles varying lengths of input data while preserving the contextual relevance of each patient’s time series measurements.

All features were standardized and normalized before being input to each machine learning and deep learning models.

1. Mechanism of weight predictor

The weight predictor layer in the proposed network adaptively weights the input features, enhancing the predictive performance by emphasizing the most relevant features for predictive task. This mechanism involves a series of operations, including dimensionality reduction weight prediction, and temporal consistency, as details below.


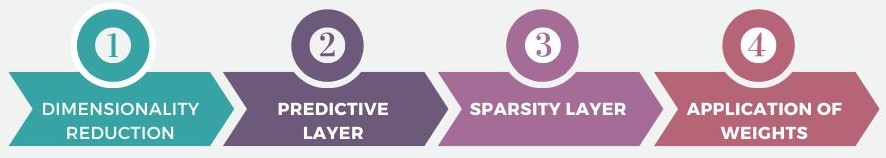


Dimensionality reduction: The first step involves reducing the dimensionality of the input while preserving their temporal significance. This is achieved by computing the mean across the time dimension using the K.mean function, which calculates the means of a tensor. The model condenses the temporal information into a single representation per feature. This summarization captures the overall importance of each feature over time.

$$r=\frac{1}{T}\sum_{t=1}^{T} x_{t}$$

where: $x_{t}$ is the value of feature at time step $t$

$T$ is the total number of time steps

$r$ denotes the single mean value for the feature

The weight prediction phase is a two-stage process that involves:

Predictive layer: A dense layer with a ‘tanh’ activation function is utilised to predicts initial weights for each feature. The choice of ‘tanh’ activation allows the model to assign both positive and negative weights, reflecting the positive or inhibitory effect of features on the predictive task.

$$w_{pred = \tanh(W_{pred}r+ b_{pred})}$$

where, $w_{pred}$is the predicted weight for each feature,

$W_{pred}$is weight parameter of the dense layer,

$b_{pred}$ is bias of the dense layer

Sparsity layer: To further refine the predicted weights and enforce feature selection, a second dense layer with a ‘sigmoid’ activation function is employed. The sigmoid activation scales the weights toa range between 0 and 1. This creates a gating mechanism that softly selects features. This layer applies a soft mask over the features, scaling their contributions based on learned importance.

$$w_{sparse}= \sigma(W_{sparse} w_{pred}+ b_{sparse})$$

where, $w_{sparse}$represent the refined weight for each feature,

$W_{sparse}$is weight parameter of the sparsity layer,

$b_{sparse}$ is bias of the sparsity layer

$\sigma$ denotes the sigmoid activation function

The resulting weight are expanded and repeated across the temporal dimension to maintain temporal consistency. This ensures that each time step receives consistent feature emphasis as determined by the weight predictor.

$$X_{weighted}=X.w_{sparse}$$

where $X_{weighted}$ is the sequence weighted by the learned importance of each feature,

$X$ is the original input sequence

1. Supplementary experiment results

Table S1: Statistical analysis of heart rate variability (HRV) features between survivors and nonsurvivors.

| Feature | Mean ± SD Survived | Mean ± SD Not Survived | p_value | AUROC |
| --- | --- | --- | --- | --- |
| SDNN (ms) | 14.92(± 5.36) | 8.37(± 1.82) | 0.01 | 0.87 |
| MeanRR (ms) | 224.98(± 37.60) | 194.42(± 14.20) | 0.05 | 0.8 |
| RMSSD (ms) | 226.57(± 37.59) | 195.48(± 14.51) | 0.04 | 0.81 |
| pNN50 (%) | 2.05(± 1.23) | 0.64(± 0.16) | 0.02 | 0.84 |
| P_VLF (s^2/Hz) | 2.18(± 1.56) | 1.18(± 1.52) | 0.12 | 0.74 |
| P_LF (s^2/Hz) | 4.14(± 2.75) | 2.60(±3.15) | 0.21 | 0.7 |
| P_HF (s^2/Hz) | 1.26(± 0.75) | 1.00(± 1.22) | 0.34 | 0.65 |
| P_VLF (%) | 37.41(± 5.81) | 38.54(± 7.98) | 0.96 | 0.48 |
| P_LF (%) | 46.59(± 3.47) | 47.24(± 6.03) | 0.44 | 0.37 |
| P_HF (%) | 15.99(± 2.93) | 14.21(± 2.26) | 0.29 | 0.67 |
| pf_VLF (Hz) | 0.02(± 0.00) | 0.01(± 0.00) | 0.11 | 0.75 |
| pf_LF (Hz) | 0.08(± 0.01) | 0.08(± 0.01) | 0.34 | 0.34 |
| pf_HF (Hz) | 0.17(± 0.00) | 0.17(± 0.00) | 0.96 | 0.51 |
| LF_HF | 3.98(± 0.5) | 4.27(± 0.55) | 0.34 | 0.34 |
| REC (%) | 54.71(± 10.23) | 60.76(± 10.91) | 0.49 | 0.38 |
| DET (%) | 80.94(± 2.91) | 77.20(± 4.57) | 0.15 | 0.72 |
| LAM (%) | 73.8(± 3.41) | 69.59(± 4.65) | 0.11 | 0.75 |
| Lmean (bts) | 24.60(± 9.29) | 34.04(± 8.75) | 0.07 | 0.21 |
| Lmax (bts) | 136.93(± 44.464) | 174.87(± 19.10) | 0.01 | 0.12 |
| Vmean (bts) | 29.51(±13.74) | 40.21(± 15.05) | 0.21 | 0.3 |
| Vmax (bts) | 111.95(±36.52) | 136.88(± 31.61) | 0.18 | 0.28 |
| SD1 | 12.79(± 5.34) | 7.14(± 1.08) | 0.04 | 0.81 |
| SD2 | 15.97(± 5.62) | 8.62(± 1.76) | 0.01 | 0.9 |
| alpha1 | 0.72(± 0.09) | 0.71(± 0.11) | 0.75 | 0.55 |
| alpha2 | 0.81(± 0.10) | 0.78(± 0.15) | 0.89 | 0.52 |

Table S2: Comparison of best cross validation performance between proposed model and other approaches.

| DL model | Train parameters | Best model layer kernel |
| --- | --- | --- |
| RNN | 22,569 | 128 |
| LSTM | 24,489 | 64 |
| BiLSTM | 16,169 | 64 |
| BiGRU | 12,841 | 32 |
| Weight-BiLSTM | 7,357 | 16 |
| ML model |  | Best hyperparameters |
| LightGBM |  | Bagging fraction = 0.8  Feature fraction = 0.6  Number of leaves = 24 |
| RF |  | Max depth = 10  Max features = sqrt  Min samples leaf = 1  Min sample split = 2  Number of estimators = 300 |
| XGBoost |  | Col sample bytree = 0.7  Learning rate = 0.01  Max depth =3  Number of estimators = 100 |
| LR |  | C = 30  Penalty = l1  Solver = liblinear |

Table S3: Comparison of performance with state-of-the-art methods on the test datasets.

| Model | Sensitivity (95%CI) | Specificity (95%CI) | PPV (95%CI) | Accuracy (95%CI) | AUROC (95%CI) | AUPRC (95%CI) |
| --- | --- | --- | --- | --- | --- | --- |
| LightGBM | 0.779 (0.5-1.000) | 0.976 (0.82-1.000) | 1.000 (0-1.000) | 0.835 (0.583-1.000) | 0.889 (0.656-1.000) | 0.944 (0.821-1.000) |
| RF | 0.449 (0.125-0.778) | 0.611 (0-1.000) | 0.798 (0.333-1.000) | 0.504 (0.25-0.75) | 0.831 (0.583-0.978) | 0.782 (0.556-1.000) |
| XGBoost | 0.776 (0.667-1.000) | 0.945 (0.5-1.000) | 1.000 (0-1.000) | 0.833 (0.583-1.000) | 0.888 (0.64-1.000) | 0.945 (0.64-1.000) |
| LR | 0.335 (0-0.667) | 0.636 (0-1.000) | 0.745 (0-1.000) | 0.417 (0.167-0.667) | 0.5 (0-1.000) | 0.768 (0.5-1.000) |
| RNN | 0.889 (0.331-1.000) | 0.309 (0-1.000) | 0.799 (0.429-1.000) | 0.748 (0.6-0.866) | 0.767 (0.641-0.926) | 0.864 (0.706-0.976) |
| LSTM | 0.555 (0.222-0.875) | 0.642 (0-1.000) | 0.715 (0.333-1.000) | 0.501 (0.25-0.75) | 0.642 (0.412-0.842) | 0.856 (0.624-1.000) |
| BiLSTM | 0.916 (0.75-1.000) | 0.642 (0-1.000) | 0.901 (0.667-1.000) | 0.914 (0.75-1.000) | 0.915 (0.75-1.000) | 1.000 (1.000-1.000) |
| BiGRU | 0.842 (0.75-0.971) | 0.75 (0.55-0.952) | 0.661 (0.333-1.000) | 0.904 (0.822-0.978) | 0.807 (0.75-1.000) | 0.988 (0.968-1.000) |
| Weight-BiLSTM | 0.917 (0.898-1.000) | 0.972 (0.86-1.000) | 1.000 (1.000-1.000) | 0.917 (0.75-1.000) | 0.926 (0.766-1.000) | 1.000 (1.000-1.000) |

Figure S1: HRV feature changes over 24 hours for survivors and nonsurvivors.


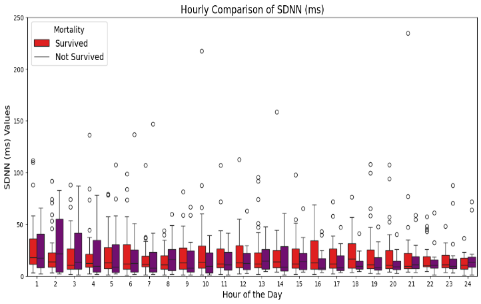

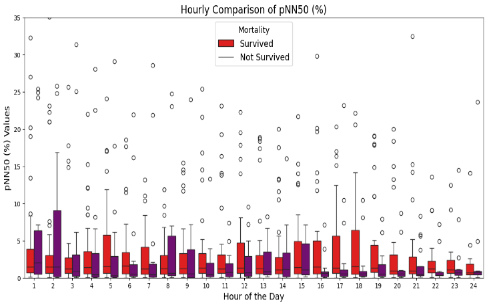

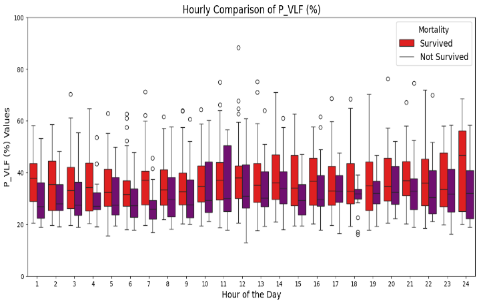

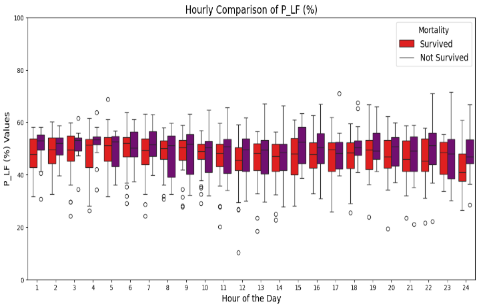

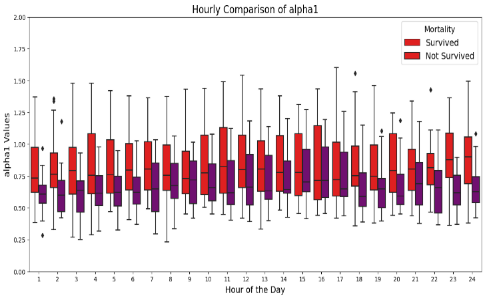

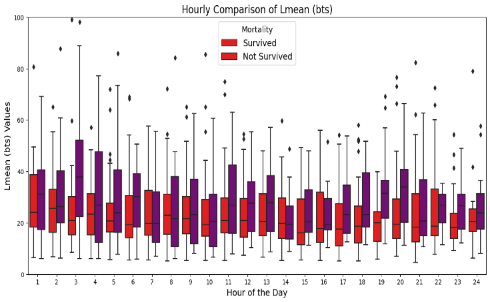

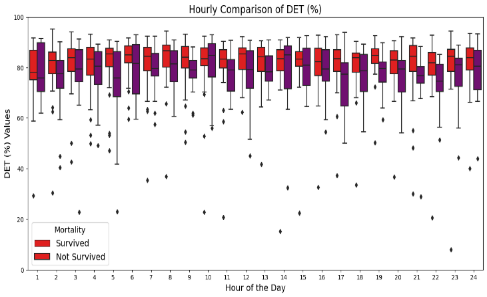

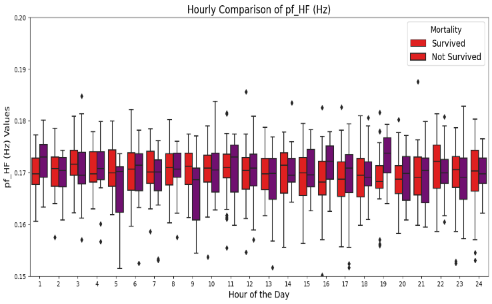

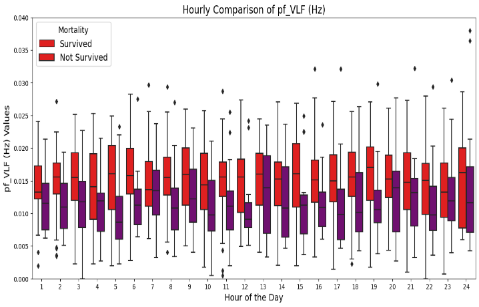


(a)

Mean validation performances

BiLSTM units kernel size


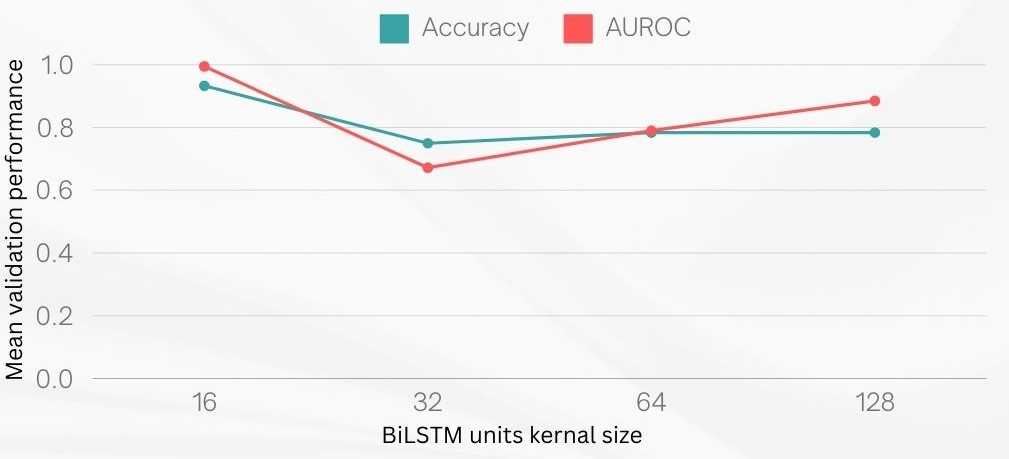


(b)


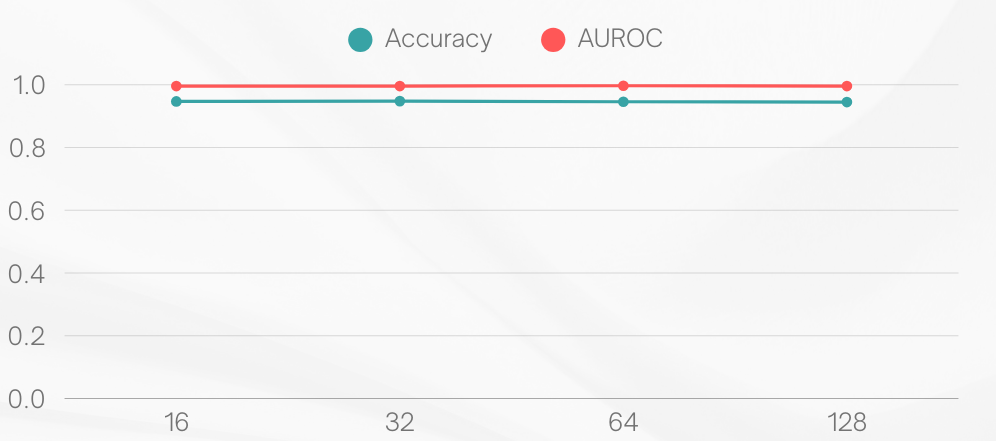


Mean validation performances

BiLSTM units kernel size

Figure S2: (a) Performance of Weight-BiLSTM with different kernel size in the 5-fold cross-validation. (b) Performance of Weight-BiLSTM with different kernel size in the Leave-One-Out subject wise cross validation (LOOCV).

Figure S3: Dynamic graph of proposed model (weight-BiLSTM) (accuracy, loss and AUROC analysis)


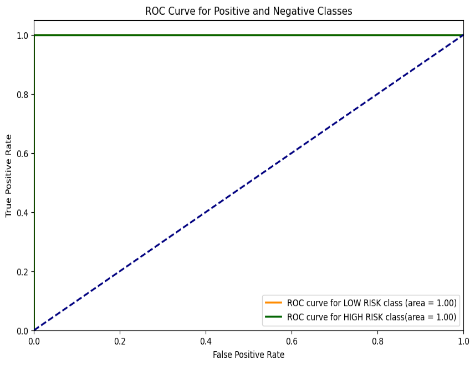


model accuracy

model loss

AUROC curve analysis


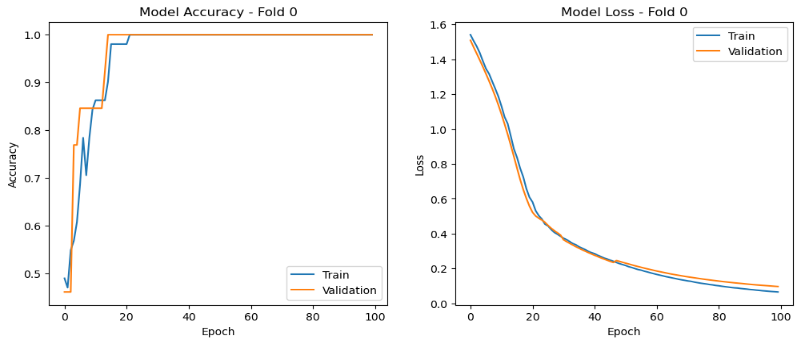


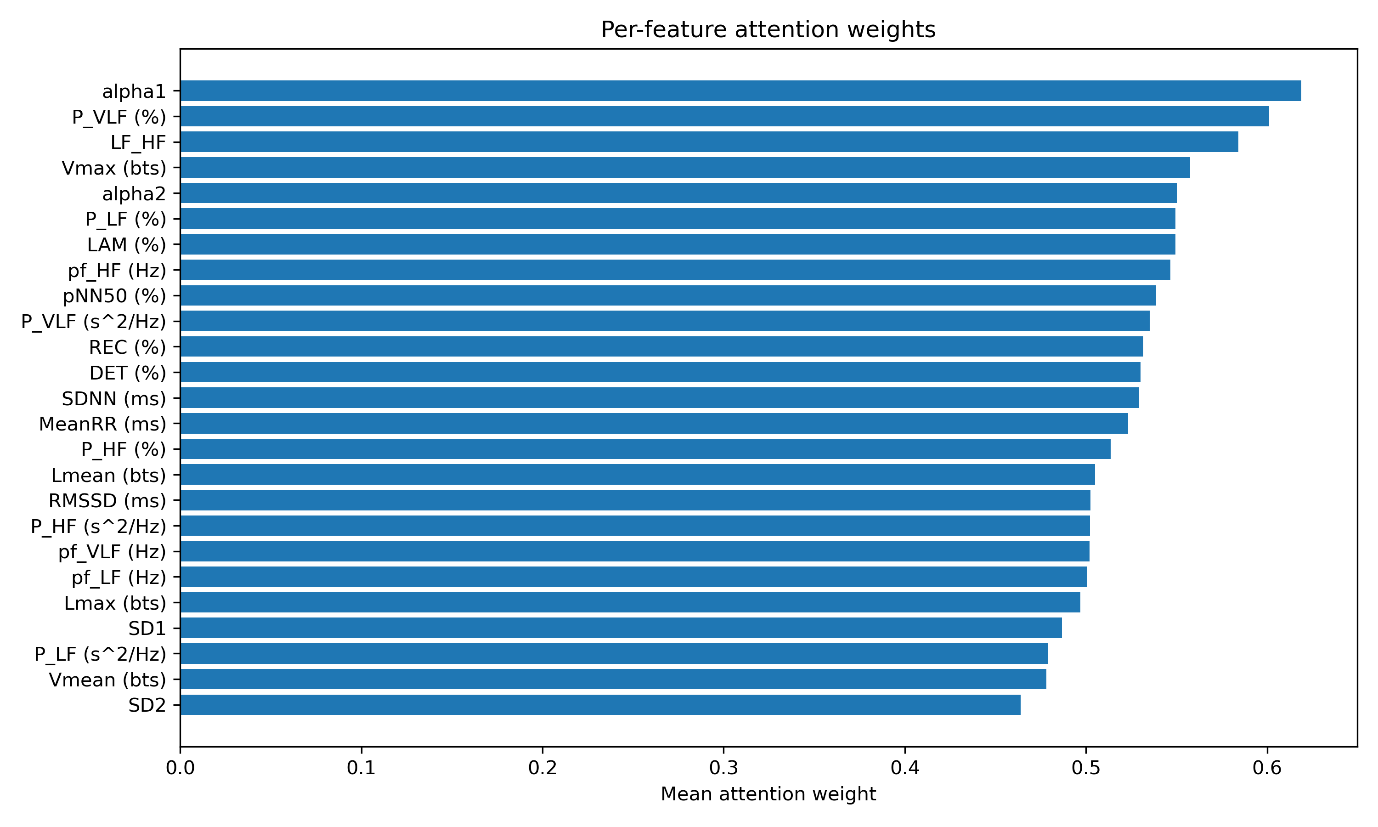


Figure S4: Mean attention weights per HRV features.


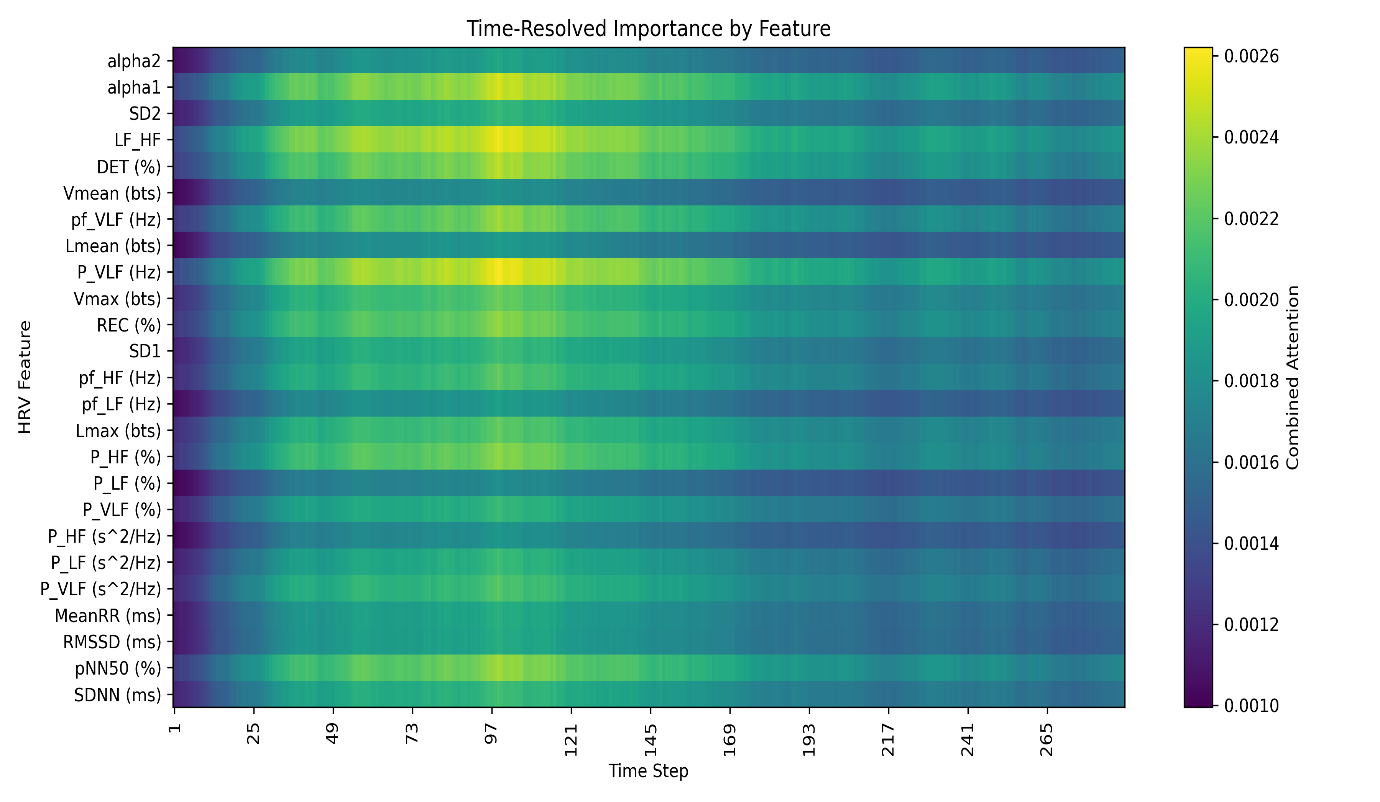


Figure S5: Time-resolved HRV features importance. Each row represents one of the 25 HRV metrics (ordered as in the main text), and each column corresponds to a 5-minute epoch (1–288).
